# An app a day: Results of pre- and post-surveys of knowledge, attitudes, and practices (KAP) regarding antimicrobial stewardship principles among nurses who utilized a novel learning platform

**DOI:** 10.1017/ash.2023.131

**Published:** 2023-03-02

**Authors:** Laura J. Bobbitt, Christo Cimino, Kim V. Garvey, Leanna S. Craft, Nicole A. Eichenseer, George E. Nelson

**Affiliations:** 1 Department of Pharmaceutical Services, Vanderbilt University Medical Center, Nashville, Tennessee; 2 Department of Anesthesiology; Center for Advanced Mobile Healthcare Learning, Vanderbilt University Medical Center, Nashville, Tennessee; 3 Department of Nursing, Vanderbilt University Medical Center, Nashville, Tennessee; 4 Division of Infectious Diseases, Department of Medicine, Vanderbilt University Medical Center, Nashville, Tennessee

## Abstract

**Background:**

Nurses perform several functions that are integral for antimicrobial stewardship (AMS). However, nurses are underrepresented in research and underutilized in implementation of AMS interventions. The objective of this pilot study was to assess the effect of asynchronous microlearning on inpatient nursing staff knowledge, attitudes, and practices (KAP) regarding AMS principles.

**Methods:**

A team of pharmacists, physicians, and nurses developed 9 case-based, multiple-choice questions with accompanying educational explanations on associated AMS principles. One case was delivered to participants daily via an institutional web-based application (QuizTime). A KAP survey with 20 questions on a 5-point Likert scale was administered before and after the intervention. Survey results were compared using a Wilcoxon signed-rank test.

**Results:**

Participants’ mean survey score after the intervention demonstrated statistically significant improvement for 18 (90%) of 20 items compared to before the intervention. Participants’ confidence improved in key AMS activities: (1) differentiating between colonization and infection (mean difference, 0.63; P < .001), (2) identifying unnecessary urine cultures and inappropriate treatment of urinary tract infections (mean difference, 0.94; P < .001), (3) recognizing opportunities for intravenous to oral therapy conversion (mean difference, 1.07; P < .001), and (4) assessing for antibiotic-associated adverse effects (mean difference, 0.54; P < .001).

**Conclusions:**

Nursing education provided through an asynchronous, microlearning format via a mobile platform resulted in statistically significant improvement in most KAP topics. Nurses are integral members of a multidisciplinary AMS team, and novel education methods can help equip them with the necessary AMS tools. This pilot study forms the basis for expanded AMS educational efforts in all healthcare professionals.

A multidisciplinary approach to antimicrobial stewardship (AMS) is necessary for success.^1,2^ Major stakeholders in AMS programs include clinicians, pharmacists, infection preventionists, microbiologists, information technology staff, and nurses. Nurses are uniquely positioned at the frontline of antibiotic administration and the center of communication among the healthcare team. Additionally, their focus on patient advocacy and safety are potential motivators for AMS involvement.^3^ Emphasis on engaging nurses in AMS efforts has increased.^4–7^ A 2017 white paper by the American Nurses Association (ANA) and the Centers for Disease Control and Prevention (CDC) highlighted daily nursing activities integral for improving antibiotic use such as obtaining early and appropriate cultures, administering antibiotics, recognizing adverse effects of antibiotics, educating patients and families, and taking appropriate allergy histories, among others.^8^

Several studies have evaluated the knowledge, attitudes, and practices (KAP) of nurses and the barriers to full nursing participation in AMS.^9–11^ Nurses are willing to be involved in AMS but are often uncertain of their role.^3^ A major barrier consistently identified is the lack of formal education on antibiotics and microbiology.^6,11–13^ Online learning modules have been successful in improving awareness of nurses’ role as antimicrobial stewards and empowering nurses to participate in clinical discussions involving antimicrobials.^14,15^

Microlearning—a form of spaced learning utilizing small units of information delivered over a short period^16^—is an emerging education strategy among healthcare professionals. Shorter and more targeted learning material is more easily processed and better retained than traditional longer formats.^17^ Microlearning has been shown to improve performance among students in health professions.^18^ However, this novel pedagogical approach has not been applied in nursing AMS education. We evaluated the effect of an asynchronous mobile microlearning platform (QuizTime) on nursing staff KAP regarding AMS principles.

## Methods

A team of physicians, pharmacists, and nurses developed a course consisting of 9 case-based multiple-choice questions. Topics included defining antimicrobial resistance and understanding nurses’ role in AMS, differentiating a urinary tract infection from asymptomatic bacteriuria, identifying characteristics of sepsis and understanding the importance of early antibiotic administration, ensuring appropriate indications prior to obtaining cultures, identifying intravenous (IV) to oral (PO) conversions, identifying opportunities for de-escalation based on culture results, taking an accurate allergy history, recognizing common adverse effects of antibiotics, and utilizing the 4 moments of antibiotic decision making.^19^

One question per day was delivered to participants via an institutional web-based application (QuizTime) and was followed by a brief educational explanation on the associated AMS principles (Supplementary Material). The course opened on December 20, 2021, and closed on February 14, 2022. Learners could enroll at any time during the rolling enrollment period and received their first question 24 hours later. Each question allowed 1 reattempt, and any unopened question was resent to learners for another attempt within 48 hours after the final question. Learners could complete the questions from anywhere, including outside the institutional network.

A KAP survey with 20 identical items on a 5-point Likert scale (1, strongly disagree, to 5, strongly agree) was administered to participants before and after the course. The latter survey was completed immediately after course conclusion. The postcourse survey also included additional questions on respondents’ confidence in their ability to participate in AMS, course effectiveness, education format preferences, and barriers to nursing participation in AMS. Survey results before and after the course were compared using a Wilcoxon signed-rank test. All statistical analyses were conducted using SPSS version 26 software (IBM, Armonk, NY).

All inpatient nurses were eligible for participation. An announcement was sent via an institutional nursing listserv, which included ∼3,000 recipients. We also recruited 2 nurse champions who assisted with participant recruitment on their respective units. Continuing nursing education (CNE) credits were awarded to participants based on an estimated completion time of 5–10 minutes per case. The study protocol and surveys were reviewed and exempted from approval by the Vanderbilt University Medical Center institutional review board.

## Results

### KAP survey

In total, 55 learners enrolled in the QuizTime course “Antimicrobial Stewardship for Nurses” from December 20, 2021, through January 30, 2022. The learners identified their specialties as cancer biology (n = 3), cardiac surgery (n = 6), medicine (n = 8), neurological surgery (n = 11), neurology (n = 9), other (n = 8), surgery (n = 5), and the Vanderbilt-Ingram Cancer Center (n = 5). Of these 55 learners, 41 (75%) completed all 9 questions and 48 (87%) completed more than half of the questions.

Of the 55 participants who completed the course, 46 (78%) completed the surveys both before and after the course. Participant characteristics are reported in Supplementary Table S1. Most of these participants (78%) held bachelor’s degrees in nursing; 56% had <5 years of experience and 39% had >10 years of experience. Also, 86% of participants reported that they administer antibiotics to >50% of their patients; 50% of respondents reported that >75% of their patients receive antibiotics. Only 2 participants (4.3%) reported attending an antibiotic-related seminar in the prior year.

The mean scores for each item on the precourse survey are presented in Supplementary Tables S2 and S3. Only 41% of participants either agreed or strongly agreed that they were familiar with the term “antimicrobial stewardship.” However, 98% were familiar with the term “antimicrobial resistance,” and 96% of participants agreed that antibiotic use can lead to antimicrobial resistance. Almost all participants (93%) agreed or strongly agreed that antibiotic use can lead to adverse drug effects. Furthermore, 82% reported understanding the relationship between antibiotics and *Clostridioides difficile*, and 87% agreed that they could usually recognize whether their patient’s change in clinical status is due to a possible infection. Also, 61% of participants felt that they had little control over what antibiotics their patients received. Additionally, 39% thought that they served as antibiotic stewards for their patients, and 43% of respondents believed that their nurse colleagues functioned as antibiotic stewards. However, 78% of those surveyed agreed that nurses should be involved in AMS. Finally, only 4% of participants were familiar with the 4 moments of antibiotic decision making.^19^

**Fig. 1.**
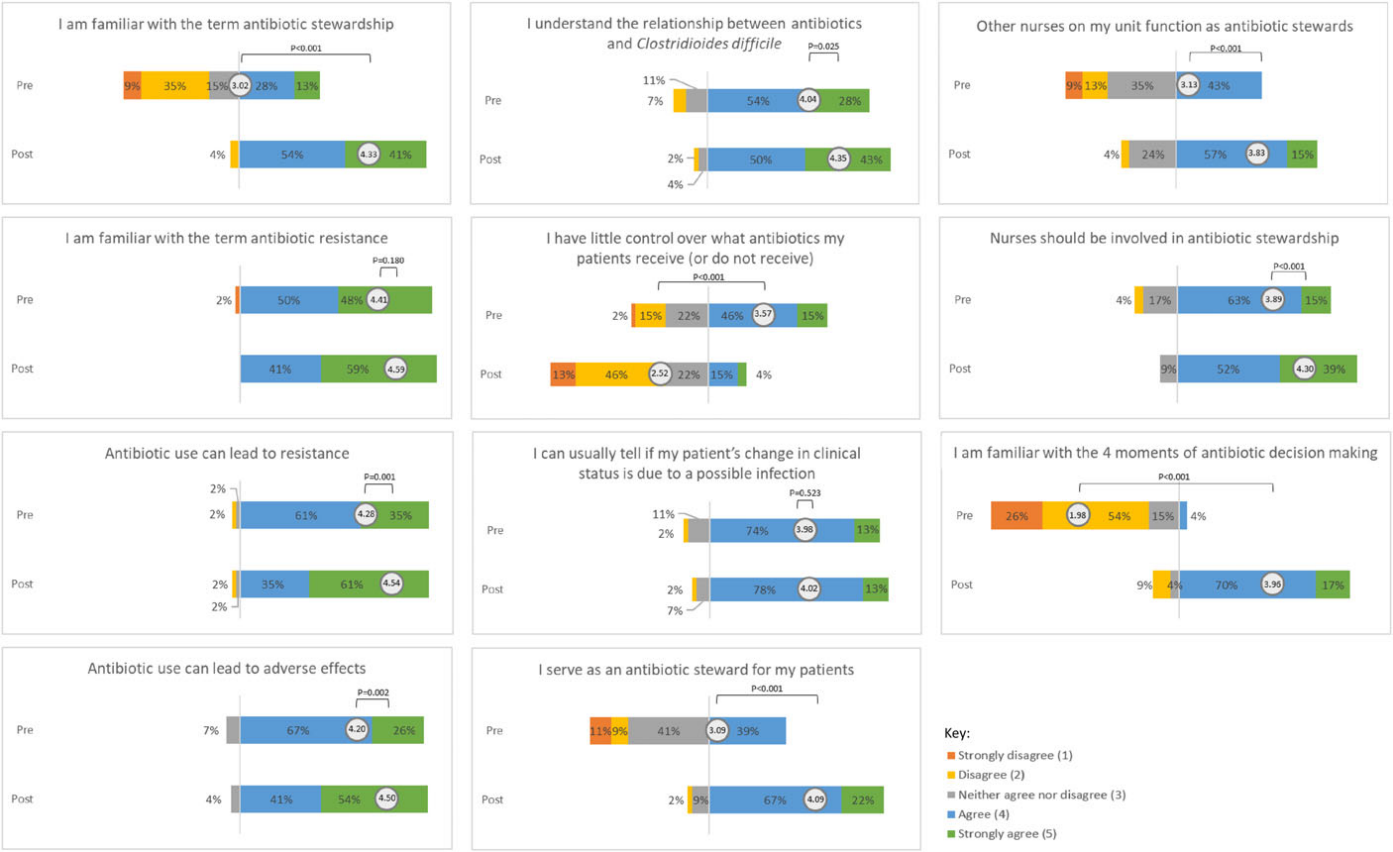
Participant knowledge, attitudes, and practices (KAP) regarding antimicrobial stewardship principles.

**Fig. 2.**
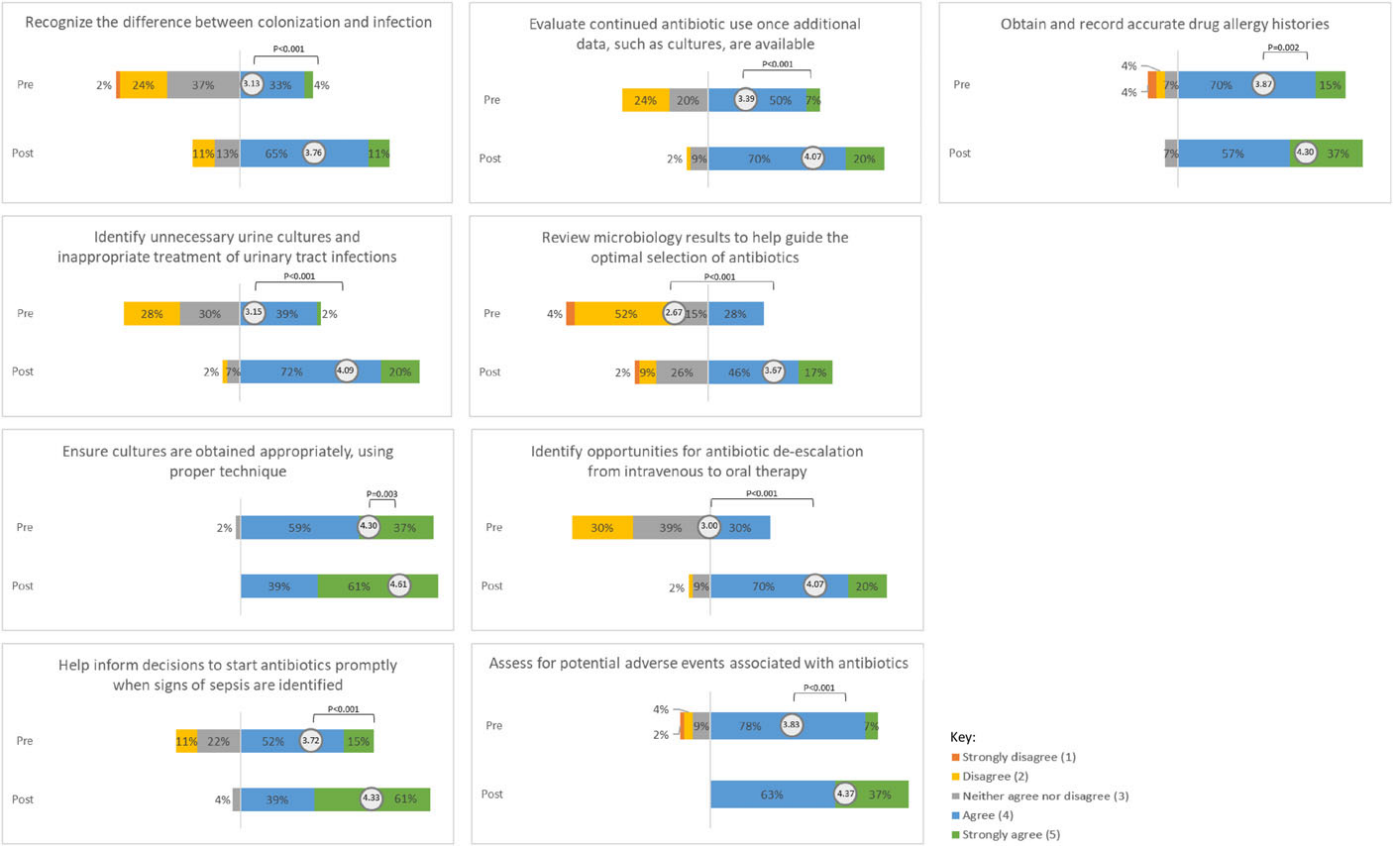
Participant confidence in ability to participate in antimicrobial stewardship activities.

The postcourse survey results were compared to the precourse scores (Figs. 1 and 2). The mean score significantly improved for 18 (90%) of 20 items. The following items showed the largest change: (1) familiarity with the 4 moments of antibiotic decision making^19^ (mean difference, 1.98; *P* < .001), (2) familiarity with the term “antimicrobial stewardship” (mean difference, 1.31; *P* < .001), (3) confidence in identifying opportunities for IV to PO conversions (mean difference, 1.07; *P* < .001), (4) beliefs that participants have little control over what antibiotics their patients receive (mean difference, −1.05; *P* < .001), (5) beliefs that participants serve as antibiotic stewards for their patients (mean difference, 1.00; *P* < .001), and (6) confidence in reviewing microbiology results to help guide optimal selection of antibiotics (mean difference, 1.00; *P* < .001). After course completion, 96% of participants felt more confident in their ability to participate in AMS, 93% of participants thought the course filled knowledge gaps about AMS, and 89% believed that the course was an effective teaching tool. Also, 70% of participants agreed that they preferred to receive education in a spaced, microlearning format rather than in a single sitting.

## Discussion

A pilot study assessing the use of asynchronous microlearning was effective in improving nursing KAP surrounding AMS. Participants demonstrated a baseline understanding of antimicrobial resistance (mean precourse score, 4.41) and antibiotic adverse effects (mean precourse score, 4.20), but larger gains were demonstrated for more specific AMS roles and responsibilities. After course completion, participants felt more confident in their ability to recognize antibiotic adverse effects, colonization versus infection, unnecessary urine cultures, and opportunities for IV to PO conversion of antimicrobials. After course completion, more nurses agreed that they have some control over what antibiotics their patients receive and believed that they could serve as antibiotic stewards.

Nurses perceive they have a role in AMS, but knowledge gaps of microbiology and antibiotic use principles are perceived barriers to integrating nurses into AMS.^6,11–13^ Carter et al^10^ reported that practices such as questioning the need for urine cultures, ensuring proper culturing technique, recording an accurate penicillin allergy history, encouraging a prompt switch from IV to PO antibiotics, and initiating antibiotic timeouts are perceived as an extension of nurses’ role. Hamdy et al^20^ conducted 12 focus groups with 90 pediatric nurses on their role in AMS. They reported that nursing roles in AMS may include advocating for the patient, communicating with the team, administering antibiotics safely, educating caregivers, and educating themselves.^20^ The role of nurses in AMS has also been endorsed by the American Nurses Association (ANA) and the Centers for Disease Control and Prevention (CDC).^8^ Nursing competency in AMS is one of the key components of a proposed AMS nursing practice framework that emphasizes the need for development of targeted AMS nursing education programs.^21^

Microlearning is an emerging approach in healthcare continuing education. This innovative pedagogy has demonstrated a positive effect on knowledge acquisition, confidence, and in some cases, clinical practice, but data on using this approach in nursing education are lacking.^18^ Some of the benefits of microlearning include the shorter time requirement and the flexibility of completing the activities at the convenience of the learner. In the age of mobile phone applications, learners tend to prefer shorter, more informal content.^22^ Microlearning may be particularly suitable for healthcare professionals, who are often managing multiple patients and tasks at one time. In addition, educational modalities, such as QuizTime, allow for scalable learning interventions in which rapid dissemination of information to large numbers of learners is possible.^16^ Once a question bank is created, the application can deploy the education to more users with little effort by study personnel. Furthermore, this approach promotes high learner engagement with sustained knowledge acquisition.^17^

Although additional education remains an important opportunity to increase nursing participation in AMS, another major barrier is prescriber pushback and challenges with interdisciplinary communication.^23^ One participant in our study stated, “Nurses are not the ones deciding on when to start antibiotics or which [antibiotics] to use. We can advocate for our patients but at times nurses can be overruled by physicians.” This sentiment was a prominent finding in a survey of 451 nurses, in which 92% of respondents reported that initiating discussions with a prescriber about antibiotic changes would benefit their patients but only 16% thought that this could be implemented without difficulty.^11^ Interdisciplinary communication is vital for successful nurse-driven AMS interventions.^7^ The SBAR (situation, background, assessment, and recommendation) tool is a successful communication style utilized in health care. A proposed AMS nursing practice framework advocates for using the SBAR to strengthen communication between nurses and providers, and the familiar SBAR tool could be adapted for use regarding AMS nursing interventions.^6^

This study had several limitations. Notably, participation in the intervention was low, which is unsurprising for a pilot study; therefore, our findings lack generalizability to other medical centers. Recruitment was challenged by nursing shortages and increased use of agency and per diem staff due to the COVID-19 pandemic. Even though any inpatient nurse was eligible to participate in the study by receipt of advertisement via an e-mail listserv, many participants joined after engagement by one of our selected nursing champions and thus our sample was not randomly chosen. Additionally, our KAP survey did not undergo reliability and validity testing. Although nurses were included in the development of the survey, we did not assess psychometric properties, which would be valuable prior to large-scale implementation. Also, we did not assess whether our intervention led to meaningful changes in clinical practice, though research has previously demonstrated changes in clinical practice following spaced education interventions.^24–27^

In this pilot study, we focused on the implementation and utility of the QuizTime platform for AMS education. Future efforts could focus on improving participation, assessing long-term impact, and scaling similar interventions to a broader population. Recruitment could be improved by targeted e-mails rather than through a listserv, repeated notifications rather than a single posting, and increased visibility throughout the institution. Future studies could measure the long-term retention of learned topics as repeated education may be needed for sustained impact. The QuizTime platform may facilitate the ease of subsequent education since the number of participants does not increase the workload for course organizers.

Nursing education and engagement in AMS provided in an asynchronous, microlearning format resulted in significant improvement in the KAP among inpatient nursing staff. Nurses are integral members of a multidisciplinary AMS team and focused AMS education may empower nurses to help reduce unnecessary antibiotic use in their patients. This pilot study supports expanded AMS educational efforts for all healthcare professionals.

## Supplementary material

For supplementary material accompanying this paper visit http://doi.org/10.1017/ash.2023.131. click here to view supplementary material
