# Supplementary material for: An app a day: Results of pre- and post-surveys of knowledge, attitudes, and practices (KAP) regarding antimicrobial stewardship principles among nurses who utilized a novel learning platform
Source: Antimicrob Steward Healthc Epidemiol. 2023 Mar 2;3(1):e41. doi: 10.1017/ash.2023.131 (PMC10028939; doi:10.1017/ash.2023.131)
Supplement: Supplementary file 1 [file S2732494X23001316sup.zip › S2732494X23001316sup002.pdf]

**Example Case**

LL is a 65-year-old male admitted with fever, chills, and altered mental status. He is found to have bacteremia and the team would like to initiate piperacillin-tazobactam. He has a penicillin allergy listed in the electronic health record that needs some clarification to ensure it is safe to administer piperacillin-tazobactam. Which of the following allergy histories MOST likely represents a true allergy?

A. Nausea/vomiting
B. Unknown reaction as a child
C. **Wheezing and shortness of breath**
D. Itching without a rash

Explanation:

About 10% of people report an allergy to penicillin. However, 95% of patients with listed penicillin allergies are not truly allergic. The most commonly reported penicillin reaction is a delayed benign rash, and these reactions may or may not recur with re-exposure. Additionally, true penicillin allergies wane over time, so patients who reacted as a child, for example, may now be tolerant of the medication. Lastly, patients may have allergies listed in the electronic health record even though their symptoms were intolerances such as diarrhea, nausea, or vomiting, which are not true allergies.

Taking a comprehensive allergy history is important because patients with a listed penicillin allergy have worse clinical outcomes and receive suboptimal treatment. Information that should be collected when taking an allergy history include:

- What happened?
- When did the reaction occur? How many years ago?
- What antibiotic?
- Was the reaction immediate (<4 hours) or delayed (>24 hours)?
- Has the patient tolerated that antibiotic or any similar antibiotics since? (similar antibiotics to penicillin include amoxicillin, ampicillin, piperacillin, cefazolin, cephalexin)
- Can the patient tolerate the antibiotic with pre-medications such as antihistamines or steroids?

| History Elements that Favor Higher Risk of Penicillin Hypersensitivity | | History Elements that Stratify as Low Risk of Penicillin Hypersensitivity |
|---|---|---|
| **Severe Delayed Symptoms at any point in the past:** | **Anaphylaxis, especially in the last 5 years:** | |
| • Mouth or Eye Ulcerations<br>• Skin or mucosal sloughing<br>• Serum sickness<br>• Immune mediated kidney Injury<br>• Immune mediated liver Injury<br>• Stevens-Johnson Syndrome (SJS)<br>• Toxic epidermal necrolysis (TEN)<br>• Drug reaction with eosinophilia and systemic symptoms (DRESS)<br>• Acute generalized exanthematous pustulosis (AGEP) | After administration of the first dose of a new treatment course with a penicillin, patient developed any of the following severe symptoms within one hour.<br>• Disseminated Hives/ Urticaria<br>• Angioedema/Swelling of Face/Throat<br>• Shortness of Breath, Wheezing, Coughing<br>• Shock<br>• Weak Pulse<br>• Loss of Consciousness/ Confusion<br>• Severe Gastrointestinal Symptoms (Diarrhea, Vomiting) | • Urticaria only, >5 years have passed<br>• Gastrointestinal symptoms only<br>• Self-limited cutaneous rash at any point<br>• Remote childhood reaction with limited details<br>• Family history of penicillin allergy only<br>• Avoidant from fear of allergy only<br>• Other symptoms, non-allergy<br>• Known tolerance of a penicillin since the original reaction occurred |

References:


- Shenoy ES, Macy E, Rowe T, Blumenthal KG. Evaluation and Management of Penicillin Allergy: A Review. *JAMA*. 2019;321(2):188-199. doi:10.1001/jama.2018.19283


**Figure S1. Example of QuizTime User Experience**

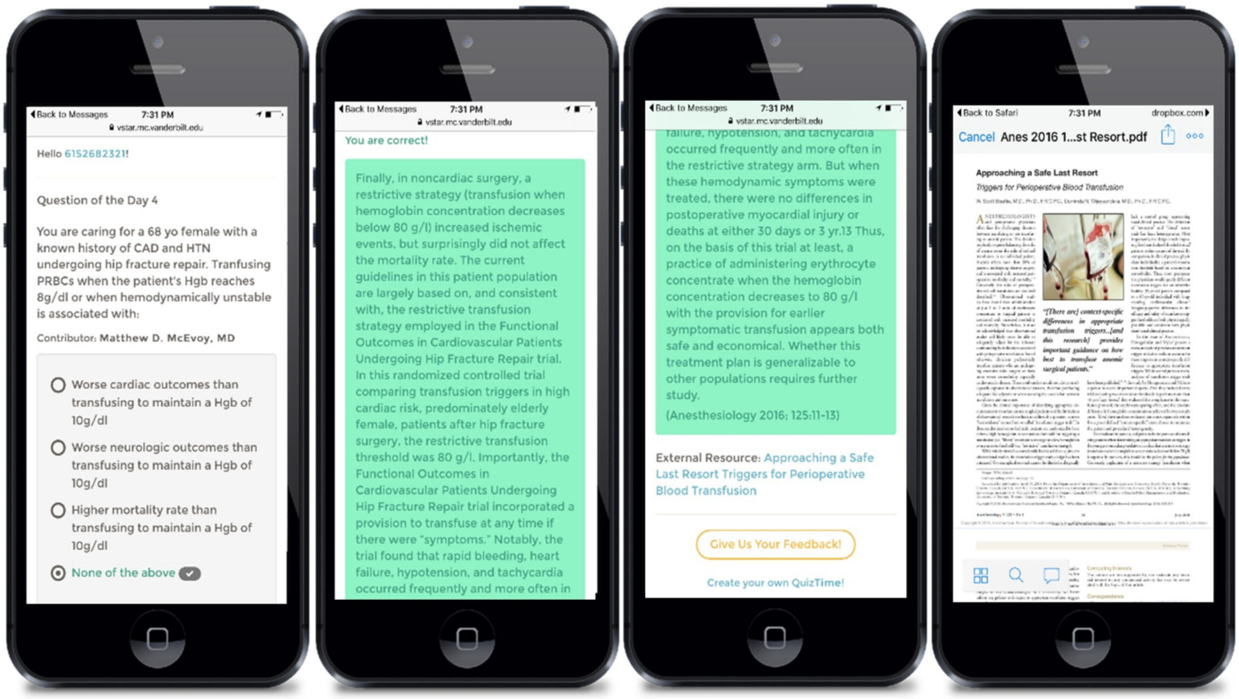

**Table S1. Knowledge, Attitudes, and Practices (KAP) Survey Participant Characteristics**

| Characteristic | No. (%) (N = 46) |
|---|---|
| **Degree** | |
| RN Diploma | 0 (0) |
| RN Associates | 3 (6.5) |
| RN Bachelors | 36 (78.2) |
| Masters | 7 (15.2) |
| Doctorate | 0 (0) |
| **Years of Experience** | |
| Less than 1 year | 6 (13.0) |
| 1-5 years | 20 (43.4) |
| 6-10 years | 2 (4.3) |
| 11-15 years | 6 (13.0) |
| More than 15 years | 12 (26.0) |
| **Shift** | |
| Day | 27 (58.6) |
| Night | 12 (26.0) |
| Both | 7 (15.2) |
| **Setting** | |
| Medical-surgical | 6 (13.0) |
| Critical care unit | 21 (45.6) |
| Progressive care/step-down unit | 14 (30.4) |
| Other | 4 (8.6) |
| **Percent of Patients Receiving Antibiotics** | |
| Less than 25% | 2 (4.3) |
| 25% to 50% | 4 (8.6) |
| 50% to 75% | 17 (36.9) |
| Greater than 75% | 23 (50.0) |
| **Attended Antibiotic-Related Seminar in Past Year** | |
| Yes | 2 (4.3) |
| No | 44 (95.6) |

**Table S2. Participant Knowledge, Attitudes, and Practices (KAP) Regarding AS Principles**

| Item | Pre-Course Mean | Post-Course Mean | Mean Difference | P-Value |
|---|---|---|---|---|
| I am familiar with the term antibiotic stewardship. | 3.02 | 4.33 | 1.31 | **<0.001** |
| I am familiar with the term antibiotic resistance. | 4.41 | 4.59 | 0.18 | 0.180 |
| Antibiotic use can lead to resistance. | 4.28 | 4.54 | 0.26 | **0.001** |
| Antibiotic use can lead to adverse effects. | 4.20 | 4.50 | 0.30 | **0.002** |
| I understand the relationship between antibiotics and *Clostridioides difficile*. | 4.04 | 4.35 | 0.31 | **0.025** |
| I have little control over what antibiotics my patients receive (or do not receive). | 3.57 | 2.52 | -1.05 | **<0.001** |
| I can usually tell if my patient's change in clinical status is due to a possible infection. | 3.98 | 4.02 | 0.04 | 0.523 |
| I serve as an antibiotic steward for my patients. | 3.09 | 4.09 | 1.00 | **<0.001** |
| Other nurses on my unit function as antibiotic stewards. | 3.13 | 3.83 | 0.70 | **<0.001** |
| Nurses should be involved in antibiotic stewardship. | 3.89 | 4.30 | 0.41 | **<0.001** |
| I am familiar with the 4 moments of antibiotic decision making. | 1.98 | 3.96 | 1.98 | **<0.001** |

1 = strongly disagree; 2 = disagree; 3 = neither agree nor disagree; 4 = agree; 5 = strongly agree

**Table S3. Participant Confidence in Ability to Participate in AS Activities**

| I am confident in my ability to: | Pre-Course Mean | Post-Course Mean | Mean Difference | P-Value |
|---|---|---|---|---|
| Recognize the difference between colonization and infection | 3.13 | 3.76 | 0.63 | **<0.001** |
| Identify unnecessary urine cultures and inappropriate treatment of urinary tract infections | 3.15 | 4.09 | 0.94 | **<0.001** |
| Ensure cultures are obtained appropriately, using proper technique | 4.30 | 4.61 | 0.31 | **0.003** |
| Help inform decisions to start antibiotics promptly when signs of sepsis are identified | 3.72 | 4.33 | 0.61 | **<0.001** |
| Evaluate continued antibiotic use once additional data, such as cultures, are available | 3.39 | 4.07 | 0.68 | **<0.001** |
| Review microbiology results to help guide the optimal selection of antibiotics | 2.67 | 3.67 | 1.00 | **<0.001** |
| Identify opportunities for antibiotic de-escalation from intravenous to oral therapy | 3.00 | 4.07 | 1.07 | **<0.001** |
| Assess for potential adverse events associated with antibiotics | 3.83 | 4.37 | 0.54 | **<0.001** |
| Obtain and record accurate drug allergy histories | 3.87 | 4.30 | 0.43 | **0.002** |

1 = strongly disagree; 2 = disagree; 3 = neither agree nor disagree; 4 = agree; 5 = strongly agree
