# Supplementary figures and images for: An app a day: Results of pre- and post-surveys of knowledge, attitudes, and practices (KAP) regarding antimicrobial stewardship principles among nurses who utilized a novel learning platform
Source: Antimicrob Steward Healthc Epidemiol. 2023 Mar 2;3(1):e41. doi: 10.1017/ash.2023.131 (PMC10028939; doi:10.1017/ash.2023.131)

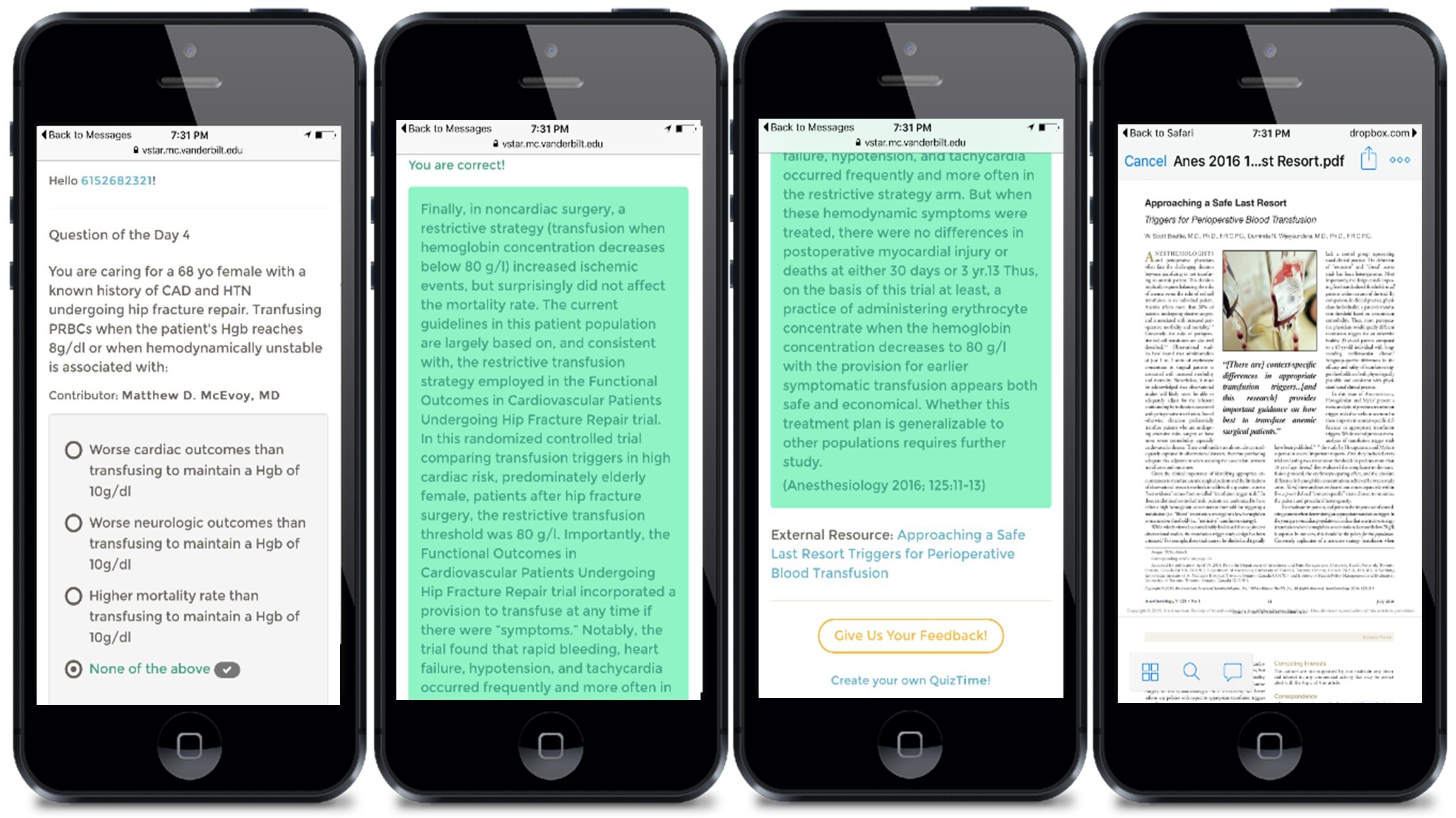

Supplement: Supplementary file 1 [file S2732494X23001316sup.zip › S2732494X23001316sup001.tiff]
